# Social Interaction With Females Modulates Context‐Dependent Male Guppy Mating Tactics for Female Receptivity

**DOI:** 10.1002/ece3.72582

**Published:** 2025-12-11

**Authors:** Versara Goberdhan, Wouter van der Bijl, Iulia Darolti, Judith E. Mank, Alberto Corral‐Lopez

**Affiliations:** ^1^ Department of Zoology and Biodiversity Research Centre University of British Columbia Vancouver Canada; ^2^ Department of Ecology and Evolution University of Lausanne Lausanne Switzerland; ^3^ Department of Ecology and Genetics Uppsala University Uppsala Sweden

**Keywords:** alternative mating tactics, male choice, mating status, sexual selection

## Abstract

Although females are traditionally viewed as the choosier sex, there is increasing evidence for the important role that male mate choice plays in sexual selection, even in species without male parental care. Social experience is a key factor influencing how individuals assess the quality of potential mates. Here, we examined how social experience shapes mating tactics and preferences in male guppies ( *Poecilia reticulata* ). Males were housed either in isolation from females or in mixed‐sex groups, and we quantified their preferences and behavioral repertoire in response to receptive and non‐receptive females in no‐choice and dichotomous choice tests. Males reared in mixed‐sex groups adjusted their mating tactics by increasing coercive behaviors toward non‐receptive females, and exhibiting shorter latencies to initiate sexual behaviors in these interactions. However, social interaction with females did not affect the overall strength of male preference for female receptivity status. While these results suggest preference for female receptivity may be shaped through interactions with other ecological factors, the observed behavioral adjustments in males reared in mixed‐sex groups align with theoretical predictions for maximizing insemination success, highlighting the key role of social experience in driving context‐dependent variation in male mating behavior.

## Graphical abstract

We experimentally tested how social experience influences male mating behavior and preference in guppies. Males raised in mixed‐sex groups adjusted their mating tactics based on female receptivity but did not differ in overall mate preference compared to males reared in male‐only groups. Our findings highlight how social experience can modulate context‐dependent mating behaviors.

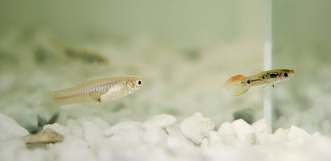

## 1 Introduction

Mate choice—any phenotypic aspect of an individual that increases the probability of engaging in sexual activity with certain individuals over others (Rosenthal 2017, after Halliday 1983)—is a central mechanism of sexual selection, shaping evolutionary trajectories across taxa (Andersson, 1994). The traditional view of females as the choosier sex, based on their typically higher reproductive investment and parental care, has evolved substantially with a growing understanding of the vast diversity of mating systems across taxa. In particular, it is clear that males also engage in mate choice, and it is well documented in taxa where males provide paternal care, such as in many pipefish and seahorse species (e.g., Rosenqvist 1990). Moreover, increasing evidence shows that male mate choice also plays a significant role in species without male parental care, where variation in female quality, sperm limitation, and the strategic allocation of mating effort can drive selective male mating behaviors (Edward and Chapman 2011; Schlupp 2021). For instance, experiments in Pacific blue‐eye fish ( *Pseudomugil signifer* ) showed that males prefer larger, more fecund females, and their choosiness shifts adaptively in response to energetic trade‐offs (Wong and Jennions 2003). However, additional investigation into the factors driving variation in flexible male mating decisions is needed, particularly in species without male parental care that make little investment during reproduction.

Social experience can play an important role in mating decision variation. The social environment provides repeated opportunities for individuals to gather information on resource availability, potential mating partners and competitors, which animals can use to adjust their behavior in ways that enhance mating success and overall fitness (Bailey and Moore 2012; Danchin et al. 2004; Fowler‐Finn and Rodríguez 2012; Valone and Templeton 2002). In species where males perform sexual displays to solicit copulation, prior social experience with females can improve a male's ability to assess female quality or to avoid soliciting females that signal unwillingness to mate (Akinyemi and Kirk 2019; Dukas 2005; Rather et al. 2022). Studies of mating systems in which males use both courtship and coercive tactics further highlight how social experience can shape mating behavior. For example, in Endler's guppies (*Poecilia wingei*), males raised in low‐competition environments increased their courtship rate as adults, while those raised with high male–male competition shifted toward more frequent coercive mating attempts (Řežucha and Reichard 2014). Similarly, in the field cricket ( *Teleogryllus oceanicus* ), males exposed to high levels of rival calling were more likely to adopt satellite behavior, positioning themselves near calling males to mate deceptively (Bailey et al. 2010). These findings underscore the importance of evaluating how the social environment shapes the plasticity of male mating strategies.

In fish, male mate choice has been documented across many species, with males often preferring female traits associated with fecundity (Schlupp 2018). For instance, males exhibit a preference for larger females, with higher fecundity potential, in species such as eastern mosquitofish ( *Gambusia holbrooki* ; Bisazza et al. 1989; Head et al. 2015; Hoysak and Godin 2007), sailfin mollies ( *Poecilia latipinna* ; Gumm and Gabor 2005), and Atlantic mollies ( *Poecilia mexicana* ; Plath et al. 2006). In fish species with internal fertilization and promiscuous, non‐resource‐based mating systems, male preference for more receptive females is expected, as such matings are more likely to result in successful insemination and higher reproductive returns per unit effort (Bonduriansky 2001; Jordan et al. 2014). This has for instance been observed in the pygmy halfbeak ( *Dermogenys collettei* ), where males preferentially associate with females exhibiting larger gravid spots, a visual marking indicative of mating receptivity status (Ogden et al. 2020).

Studies of male mate choice in guppies ( *Poecilia reticulata* ) reveal that males preferentially associate with larger females (Corral‐López et al. 2018; Dosen and Montgomerie 2004; Herdman et al. 2004; Jeswiet et al. 2012), and that they show higher courtship rates with receptive compared to non‐receptive females, and with virgins compared to non‐virgins (Guevara‐Fiore et al. 2009, 2010). Additionally, prior mating experience can influence both subsequent mating tactics and male choosiness. For example, males previously successful with receptive females reduced their courtship and increased their coercive insemination attempts, and also showed stronger preferences for larger females (Guevara‐Fiore and Endler 2018). Such plasticity in choosiness is consistent with theoretical expectations of prudent allocation of effort and limited resources, as directing investment toward high‐quality mates may optimize reproductive efficiency in variable ecological contexts (Edward and Chapman 2011; Kelly and Jennions 2011). Despite extensive research in this long‐standing sexual selection model system, guppies offer the additional opportunity to investigate how social experience shapes male preference and assessment of female receptivity.

Here, we investigated whether social experience influences male guppy preference and mating effort in response to female mating status. To test this, we isolated males from females before reaching sexual maturity and subsequently housed them in either mixed‐sex or male‐only groups. We then quantified their preference and sexual behavior repertoire in trials involving receptive and non‐receptive females. Behavioral measurements, obtained via a combination of behavioral scoring and automated fish motion tracking, incorporated both no‐choice and dichotomous choice tests to mitigate potential biases associated with different testing paradigms, such as potential overestimations of preference strength in dichotomous choice designs in species where sequential mating encounters are more typical (Dougherty and Shuker 2015). Based on theoretical expectations for fitness maximization, we predicted that males would increase their preference strength for receptive females following a long‐term exposure treatment in mixed‐sex rearing groups. Additionally, we expected that the wider range of social interactions experienced during a mixed‐sex rearing treatment would facilitate flexibility in adjusting mating tactics. Specifically, we predicted that mixed‐sex reared males would exhibit larger rates of coercive behaviors toward non‐receptive females and courtship displays toward receptive females than those reared in male‐only groups.

## 2 Methods

### 2.1 Study System

All guppies used in this experiment originated from a laboratory‐adapted stock population, originally collected from the high predation region of the Quaré River (Trinidad and Tobago) in 1998 (Pélabon et al. 2014). The population has since been maintained in large aquaria (> 200 individuals each) to minimize inbreeding. Aquaria contained gravel, water filters, and aquatic plants, and all experiments were approved by institutional animal ethics protocols. Fish were raised at a water temperature of 25°C with a 12:12 light:dark schedule, and fed a daily diet of flake food (Hikari Fancy Food) and live *Artemia* brine shrimp.

### 2.2 Experimental Procedure

We collected newborn guppies from stock aquaria and housed them in nursery tanks until they could be sexed by the development of a gonopodium, a modified anal fin (Houde 1997; Liley 1966). At this time point, males were transferred to male‐only tanks in groups of seven individuals, with no physical or visual contact with females. At approximately 4 months of age, we randomly assigned males to one of two experimental treatments: (i) *mixed‐sex rearing*, housed in mixed‐sex groups allowing for social interactions with females (three males and four females—two virgins and two mated matched for age and size); (ii) *male‐only rearing*, kept in male‐only groups. The female‐biased sex ratio in the mixed‐sex rearing treatment (≈0.40 proportion males) reflects typical adult sex ratios in natural *P. reticulata* populations (Arendt et al. 2014). Treatments lasted 45 days, allowing for variation in female receptivity cues experienced, during which fish were fed ad libitum in both treatments to mitigate potential differences in foraging competition due to the presence of females.

Approximately one‐third of males (*n* = 62) were assigned to dichotomous choice tests to assess changes in preference for receptive females. These males were tested twice: first before social treatment assignment (pre‐treatment test) to establish individual baseline preference; and a second time after the 45 days in their social treatment (post‐treatment test). Although the pre‐treatment test involved a brief (20 min) interaction with females, it provided a shared minimal exposure across treatments and was critical for quantifying baseline preference.

The remaining fish participated only in no choice tests after the 45‐day treatment, with individuals randomly assigned to interact with either a receptive (*n* = 62), or a non‐receptive female (*n* = 60). To ensure all males were of similar age during behavioral testing, and due to logistical constraints, we performed the experiment in two batches of equal numbers of individuals.

### 2.3 Dichotomous Choice Preference Tests

#### 2.3.1 Experimental Setup

To assess differences in preference for receptive females between male‐only reared and mixed‐sex reared males, we measured the time that reproductively mature males spent associating with receptive versus non‐receptive females in dichotomous choice tests. Each male underwent two tests, one before treatment assignment (pre‐treatment), and again after completing the 45‐day social exposure period (post‐treatment). We obtained pre‐treatment and post‐treatment data for 31 males per social treatment condition. Receptive and non‐receptive females allocated to the same assay were matched for body size, differing by no more than 5 mm in standard length (max difference ≈ 2%). To achieve this, same‐age females were reared since birth under standardized conditions (groups of eight, ad libitum feeding), and shortly before the experiments we measured their standard length in a small aquarium with affixed measuring tape.

We photographed each male after behavioral testing in the pre‐treatment test using a Canon EOS Rebel T7i camera and 100 mm macro lens. These photographs, together with direct visual inspection in their holding tanks, where they were housed with only a few other conspecifics, allowed us to successfully re‐identify each male before the post‐treatment test as they were transferred into a 1.7 L aquarium 3 days prior to the second dichotomous choice test. This isolation period, performed equally for both social treatment groups, allowed for sperm replenishment and mitigated biases in motivation to mate (Pilastro et al. 2002).

#### 2.3.2 Behavioral Assays and Data Collection

We performed all behavioral tests in a circular arena (diameter = 47 cm) sheltered to prevent disruption. We filmed the arena for 15‐min periods using an OBSBOT webcam (1080 P at 30 fps) after a five‐minute acclimatization period. For accurate identification of fish with tracking software, we placed them in the experimental arena in 20 s intervals. We placed females first in the arena, randomizing the order of placing receptive and non‐receptive females. Each male was tested with different receptive and non‐receptive females and water was changed in the arena between tests. To minimize stress, each fish was netted, placed in a glass bowl and transferred to the testing apparatus. For consistency, the tests were always conducted in the morning for a period of 4–6 h.

**FIGURE A1:**
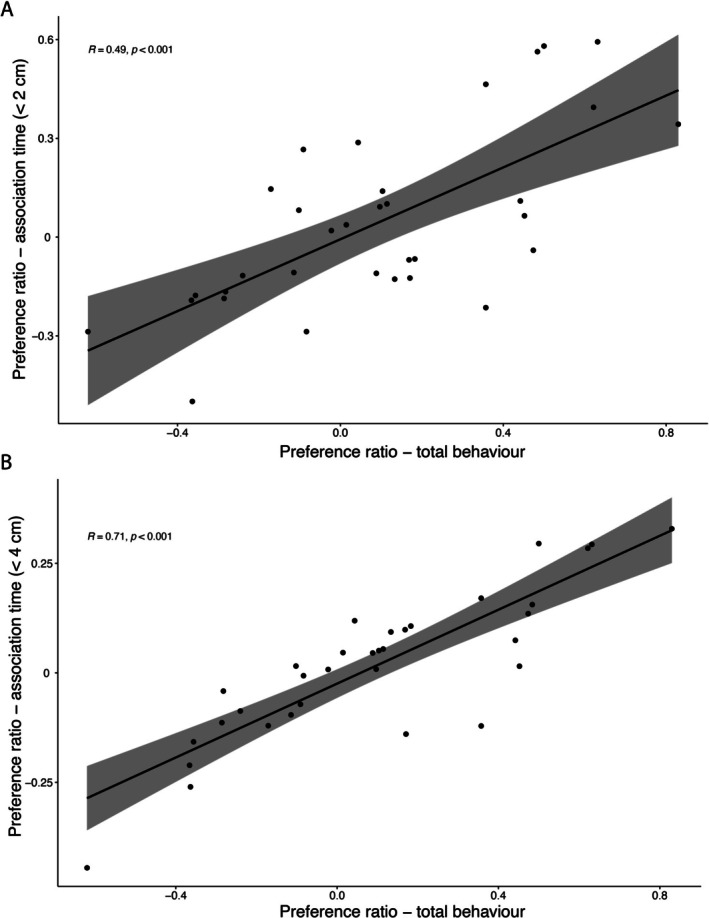
Correlation between proximity‐based preference and behavior‐based preference ratios in male guppies. Scatter plots show the relationship between preference ratios based on the proportion of time males spent at a distance < 2 cm (A) or < 4 cm (B) from each female, and the proportion of total sexual behaviors directed toward each female. Points represent each trial (*n* = 33), and solid lines the best‐fit linear regression with a 95% confidence interval around the estimated mean relationship (shaded areas).

We used idTracker (Pérez‐Escudero et al. 2014) to extract positional data from video recordings and to calculate frame‐by‐frame distances between males and each female. Prior to performing behavioral assays in this study, we validated the use of proximity‐based measurements as a proxy for male preference using data collected in prior work (see Appendix 1). Briefly, data analyses from similar assays of a separate laboratory population found over 70% correlation between preference scores obtained from the proportion of time at distances lower than 4 cm with either female, and the proportion of sexual behaviors directed to each female (Figure A1). Based on this prior work, male association time was defined as the number of video frames in which the male was within 4 cm (approximately two individuals' body lengths) of each female and we calculated a preference ratio for the receptive female in each trial as:

$$\frac{\left(\text{time spent with receptive}-\text{time spent with non}‐\text{receptive}\right)}{\text{total amount of time spent with both females}}$$

### 2.4 No Choice Preference Tests

#### 2.4.1 Behavioral Assays and Data Collection

To assess mating effort and adjustments of sexual behavior in males with different social experience toward females under varying receptivity status, we performed no‐choice tests with receptive and non‐receptive females with mixed‐sex reared and male‐only reared males. Using a different set of males from those used in dichotomous choice assays, we performed no‐choice assays with 66 mixed‐sex reared males (*n* = 34 with non‐receptive females; *n* = 32 with receptive females) and 60 male‐only reared males (*n* = 30 with non‐receptive females; *n* = 30 with receptive females). We followed the protocol described for dichotomous choice tests, except only one female was present for each test, with the female placed prior to the male in the testing apparatus. Females were also only used once and water was changed between each test.

A single observer (V.G.) blindly scored male sexual behavior in video recordings (random order based on uninformative video id). We scored the following behaviors as defined in Liley (1966): (i) frequency of *sigmoid displays*, defined as every instance in which a male positioned himself in front of the female with an S‐shaped posture soliciting copulation; (ii) frequency of *sneak attempts*, defined as unsolicited attempts at inseminating a female from behind by thrusting the gonopodium at the female's urogenital pore. From these measures, we calculated *behavioral preference*, the combined number of displays and sneak attempts directed toward each female, and *latency* to first sexual behavior.

We further used positional tracking data to quantify: (i) *preference* based on association time, measured as the number of video frames in which the male was within 4 cm of the female, and (ii) *time spent chasing*, measured as the number of frames in which the male was oriented behind the female, with nearly parallel movement directions (male–female angle < 50°), and an inter‐individual distance < 3 cm. Finally, we estimated body size data for each individual using tracking data, calculating mean body length as the average diagonal of all bounding boxes corresponding to that individual's blobs. Assays with low tracking quality (< 80% tracked frames) were disregarded for statistical analyses. The final data set included a total of 105 assays (*n* by treatment: Mixed‐sex_receptive_ = 24, Mixed‐sex_non‐receptive_ = 29, Male‐only_receptive_ = 26, Male‐only_non‐receptive_ = 26).

### 2.5 Female Receptivity

To study the role of social experience in the preference and behavior of males toward females differing in mating receptivity, we exposed males reared in mixed‐sex or in male‐only groups to receptive and non‐receptive females in dichotomous and no‐choice tests. Female receptivity in guppies is tightly linked to the reproductive cycle. Receptiveness is highest immediately after parturition for about 3 days in which new ova are fertilized, then declines linearly for the following days until it reaches a minimum approximately 10 days post‐parturition, remaining low until parturition of a new clutch of offspring (approximately 28 days; Liley 1966; Houde 1997). Receptiveness in virgin females presents a similar pattern during their first reproductive cycle (Houde 1997). Following methods in Guevara‐Fiore et al. (2010), we housed small groups of virgin females with males in a 1:1 ratio and used them in behavioral tests either the following day (receptive females) or 14 days later (non‐receptive females). Because females were continuously exposed to males in these holding groups, we expected them to be inseminated rapidly, ensuring they represented their respective receptivity treatments. Previous work using this method found clear behavioral differences between receptive and non‐receptive females (Guevara‐Fiore et al. 2010).

To validate female receptivity categories in our experiment, we quantified female behavior during pre‐treatment dichotomous choice trials, where receptive and non‐receptive females were exposed simultaneously to the same male stimuli. Using positional tracking data, we quantified female glides, defined as smooth approaches toward the male when the female was within 60 mm, moving at speeds between the 5th and 20th percentile of her speed distribution, and oriented toward the male (male–female angle ≤ 70°). Events occurring within 3 s of one another were considered as one. We used this proxy of gliding behavior to compare relative differences between female receptivity treatments for the same male stimulus. Specifically, we calculated, for each male, the difference in the number of glides performed by receptive versus non‐receptive females. This difference was significantly greater than zero (one‐sample *t*‐test: ∆glides_Receptive − Non‐receptive_ [95% CIs] = 1.76 [0.48, 3.03], *t* = 2.76, df = 62, *p* = 0.007), supporting the validity of our receptivity categorization.

### 2.6 Morphological Measurements

To assess potential differences in morphology or coloration between males across treatments, we quantified melanic and carotenoid coloration, body size, and tail size using an image‐analyses pipeline developed in the lab for an artificial selection experiment. Briefly, this pipeline uses neural network‐based image segmentation to isolate fish from photographs, align each individual's body shape to a reference shape using morphological landmarks, and quantify traits of interest from the aligned images (van der Bijl et al. 2025).

### 2.7 Statistical Analyses

All analyses were performed in R (v. 4.4.3; R Core Team 2024) using the *stats* package for linear models, the *lme4* package for Linear Mixed Models (LMMs; Bates et al. 2007), and the *glmmTMB* package for generalized linear mixed models (GLMMs; Brooks et al. 2017). Significance of fixed effects was computed using conditional *F* tests with Kenward‐Roger approximation for degrees of freedom in LMMs, and Type III Wald *χ* ^2^ tests for GLMMs implemented in *lmerTest* and *car* packages, respectively (Kuznetsova et al. 2017; Fox and Weisberg 2019). Model coefficients and confidence intervals were extracted using the *parameters* and *merDeriv* packages (Lüdecke et al. 2020; Wang and Merkle 2018). We evaluated the adequacy of our fitted models using scaled‐residuals quantile‐quantile plots, residual versus predicted values plots, and overdispersion and zero‐inflation tests in the *DHARMa* package (Hartig 2018). Post hoc comparisons of male responses between female receptivity levels, were performed using the *emmeans* package, with Tukey‐adjustment for multiple comparisons (Lenth et al. 2019).

#### 2.7.1 Dichotomous Choice Tests

To evaluate differences in preferences for receptive females between males reared in mixed‐sex and male‐only groups, we used two complementary analyses. First, we fitted an LMM with preference ratio as the dependent variable, and time of testing, social experience, and their interaction as fixed factors. Male ID was included as a random intercept to account for repeated measures on the same individual. Experimental cohort (batch) was initially included as a random factor, but was not included in the final model due to singularity issues caused by low variance in batch effects. This approach accounts for the non‐independence of observations from the same male. However, because all individuals were untreated at the pre‐treatment time of testing but still associated with a treatment group, including pre‐treatment data in the same model could bias estimates of treatment effects. Therefore, we conducted a second analysis using only data at the post‐treatment time of testing. We used an LMM with social treatment as a fixed effect and batch as a random intercept. These two analyses provided consistent results, with no significant effect of social treatment on male preference (see reporting of both approaches in Section 3).

#### 2.7.2 No‐Choice Tests

Following the experimental treatment, we compared sexual behavior in males reared in mixed‐sex and male‐only groups during no‐choice tests with receptive or non‐receptive females. We analyzed time associating with the female fitting an LMM. For sigmoid displays, sneak attempts, total time chasing and total behavior we fit GLMMs using a negative binomial distribution and a log link function for the conditional mean. Fixed factors included female mating status, male social experience and their interaction. All models also included the number of female gliding approaches and the difference in body size between females and males, estimated from tracking data, to control for their potential effect in driving variability in male sexual behavior. We additionally included the rearing tank and batch as random factors in all models. For the model of time spent chasing, an intercept‐only zero inflation term significantly improved model fit.

We additionally modeled latency to first sexual behavior using a mixed‐effect survival test performed with the *coxme* package (Therneau and Lumley 2015), with an analogous model structure. The significance of effects in this survival model was tested using likelihood ratio tests comparing models without the tested effect to the full model structure.

#### 2.7.3 Color and Morphology

We used linear models with each morphological trait as the dependent variable and social experience treatment as a fixed effect. Males randomly assigned to mixed‐sex reared and male‐only reared treatments used in dichotomous choice tests did not differ in coloration or morphological traits (see Appendix 2). These traits were not considered further for comparisons of sexual behavior across treatments.

## 3 Results

### 3.1 Dichotomous Choice Preference Tests

**FIGURE 1:**
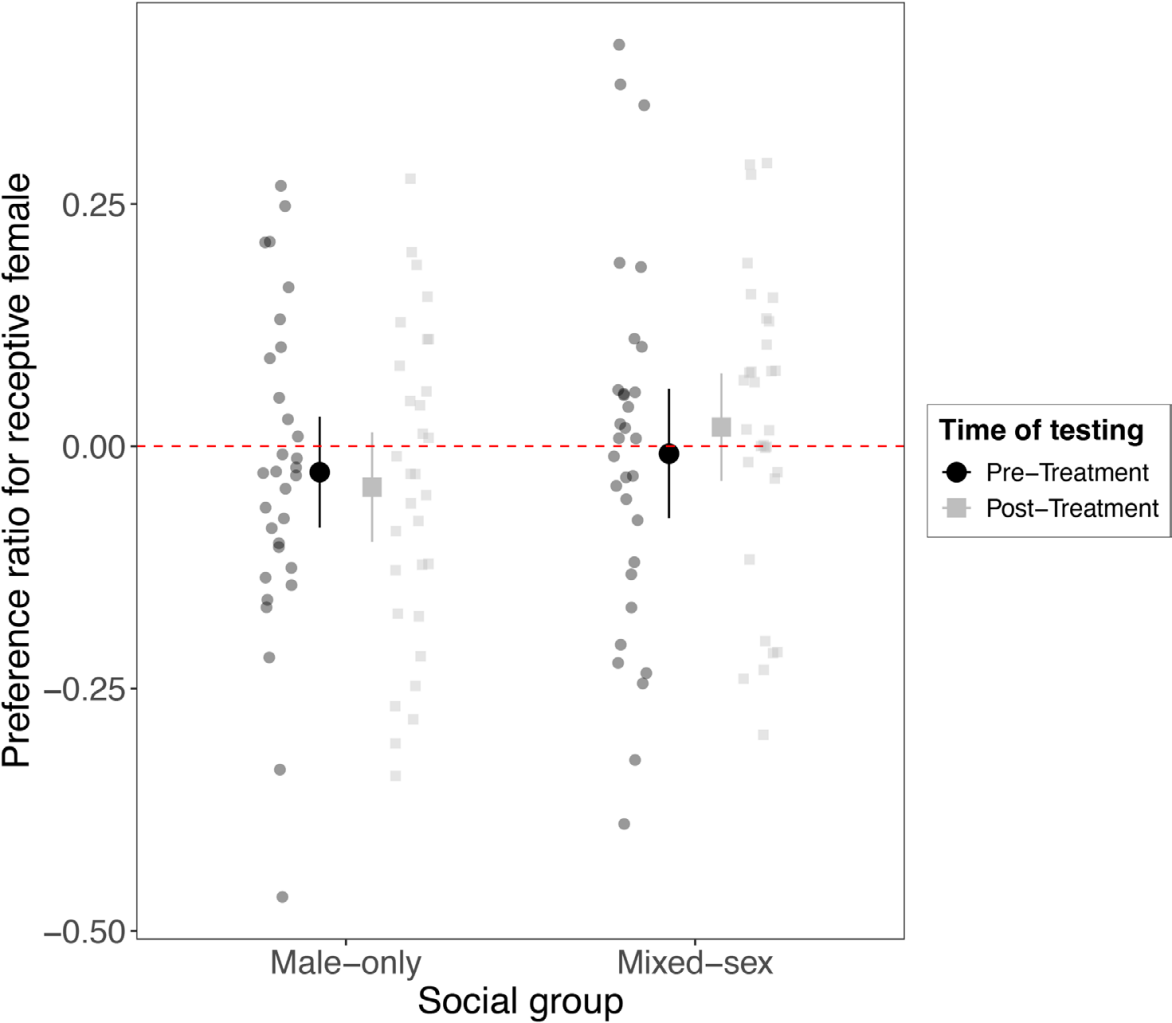
Effect of social experience in guppy male preference for receptive females. Preference ratios were calculated as the total time spent associating with the receptive female relative to the total time spent with both receptive and non‐receptive females in dichotomous choice tests. Tests were performed before (pre‐treatment, black circles) and after (post‐treatment, gray squares) a 45‐day rearing treatment in male‐only (*n* = 31) or mixed‐sex groups (*n* = 31). Larger symbols represent mean preference ratios with 95% CI bars. We found no significant differences in preference for receptive females between male‐only and mixes‐sex reared males at either time point (see Tables 1 and 2).

Analyses including both pre‐ and post‐treatment data indicated no significant difference between mixed‐sex and male‐only reared treatments on preference for receptive females (estimate_mixed‐sex_ = 0.02 ± 0.04, *t*_df = 118_ = 0.45, *p* = 0.66; Figure 1; Table 1), nor in the rate of change in preference between males exposed to these different social groups for 45 days (estimate_pre‐treatment × mixed‐sex_ = 0.04 ± 0.06, *t*_df = 118_ = 0.72, *p* = 0.47; Figure 1; Table 1). The time of testing showed also no effect in the observed preference for receptive females (estimate_pre‐treatment_ = −0.02 ± 0.04, *t*_df = 118_ = −0.37, *p* = 0.71; Figure 1; Table 1).

**TABLE 1:** Results from a linear mixed model testing for differences in guppy male preference for receptive females before and after a 45‐day treatment period in male‐only (*n* = 31) or mixed‐sex (*n* = 31) groups.

Parameter	Coefficient [95% CI]	SE	*t*_df = 118_	*p*
*Fixed effects*				
Intercept	−0.03 [−0.09, 0.03]	0.03	−0.88	0.38
Male social treatment (mixed‐sex)	0.02 [−0.07, 0.10]	0.04	0.45	0.66
Time of testing (pre‐treatment)	−0.02 [−0.10, 0.07]	0.04	−0.37	0.71
Male social treatment: time of testing	0.04 [−0.07, 0.16]	0.06	0.72	0.47
*Random effects*				
SD (Intercept: Male ID)	0.03 [0.00, 1.24]	0.06		
SD (Residual)	0.17 [0.14, 0.20]	0.02		

*Note:* The model includes social treatment, time of testing, their interaction, and experimental batch as fixed effects. Male ID was included as a random intercept to account for repeated measures on the same individual. Estimates are unstandardized coefficients.

When analyzing post‐treatment data independently, we consistently showed that preference for receptive females did not differ between males reared in mixed‐sex or male‐only groups (estimate_mixed‐sex_ = 0.02 ± 0.04, *t*_df = 58_ = 0.44, *p* = 0.66; Figure 1; Table 2).

**TABLE 2:** Results from a linear mixed model testing for differences in guppy male preference for receptive females following a 45‐day treatment period in male‐only (*n* = 31) or mixed‐sex (*n* = 31) groups.

Parameter	Coefficient [95% CI]	SE	*t*_df = 58_	*p*
*Fixed effects*				
Intercept	−0.03 [−0.12, 0.07]	0.05	−0.58	0.56
Male social treatment (mixed‐sex)	0.02 [−0.07, 0.11]	0.04	0.44	0.66
*Random effects*				
SD (Intercept: batch)	0.05 [0.01, 0.34]	0.05		
SD (Residual)	0.17 [0.14, 0.21]	0.02		

*Note:* The model includes social treatment as a fixed effect and experimental batch as a random effect. Estimates are unstandardized coefficients.

### 3.2 No Choice Preference Tests

**FIGURE 2:**
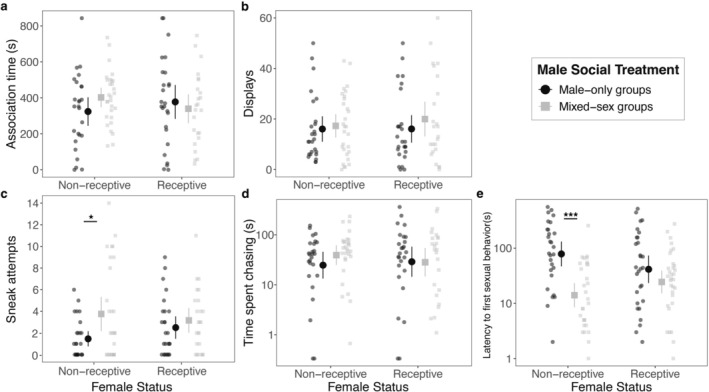
Effect of social experience with females in male sexual behavior. (a) Time spent associating with the female (inter‐individual distance < 4 cm), (b) number of sigmoid displays, (c) number of sneak attempts, (d) total time spent chasing females (s), and (e) latency to first sexual behavior (s), measured toward non‐receptive and receptive females. Behaviors were observed in guppy males following a 45‐day rearing treatment in male‐only groups (black circles, *n* = 52) or mixed‐sex groups (gray squares, *n* = 53). Larger symbols indicate mean values with 95% CI bars. Asterisks denote significant post hoc contrasts of male response between female receptivity levels across social treatment (*p < 0.05; ***p < 0.001; see Tables 3 and 4). *Y*‐axis for time spent chasing and latency are log‐transformed for easier visualization.

Similar to data obtained from dichotomous choice assays, preference based on behavioral scoring (combined display and sneak attempt frequency) significantly correlated to preference quantified from proximity‐based measurements obtained from fish tracking data (Spearman correlation test: *ρ* = 0.34, *p* < 0.001). Statistical models fitted independently for both variables consistently indicated no effect of social rearing in mixed‐sex groups in male guppy preference (LMM time associating _social treatment_: *F*_df = 1/53.4_ = 0.48, *p* = 0.49; GLMM total behavior _social treatment_: *χ*_df = 1_ = 0.82, *p* = 0.36; Figure 2a; Table 3).

**TABLE 3:** Statistical results from mixed models assessing two metrics of male guppy preference for receptive females in no‐choice tests: (i) association time (defined as distance to female < 4 cm), and (ii) total sexual behavior directed toward the female (frequency of displays + sneak attempts).

Trait	Parameter	IRR [95% CI]	SE	*z*	*p*
Total sexual behavior	Intercept	17.09 [12.38, 23.60]	2.81	17.24	**< 0.001**
	Female status (receptive)	1.08 [0.72, 1.62]	0.22	0.38	0.70
	Male social treatment (mixed‐sex)	1.20 [0.81, 1.78]	0.24	0.91	0.36
	Body size difference	1.06 [0.92, 1.23]	0.08	0.79	0.43
	Female gliding frequency	1.00 [0.98, 1.03]	0.01	0.22	0.83
	Female status: male social treatment	0.99 [0.56, 1.74]	0.29	−0.25	0.96

Trait	Parameter	Coefficient [95% CI]	SE	*t*_df = 96_	*p*
Association time	Intercept	310.1 [199.5, 420.6]	55.7	5.57	**< 0.001**
	Female status (receptive)	42.7 [−40.0, 125.5]	41.7	1.02	0.30
	Male social treatment (mixed‐sex)	77.9 [−24.8, 180.7]	51.8	1.51	0.13
	Body size difference	−34.5 [−71.2, 2.21]	18.5	−1.87	0.07
	Female gliding frequency	3.80 [−2.79, 10.40]	3.32	1.14	0.27
	Female status: male social treatment	−94.6 [−210.8, 21.6]	−58.5	−1.62	0.11

*Note:* Fixed effects included female receptivity status and male social treatment (Mixed‐sex: *n* = 52; Male‐only: *n* = 53). Random intercepts were fitted for experimental batch and rearing tank. Body size and female behavioral response were included as covariates. The model for total sexual behavior was fit using a negative binomial distribution, with estimates reported as incidence rate ratios (IRR). The model for association time was fit with a Gaussian distribution, with estimates reported as unstandardized coefficients. Significant *p*‐values (< 0.05) are shown in bold.

There was no significant difference between males reared in mixed‐sex and male‐only groups in their average levels of display behavior, nor in the mean number of displays that were performed toward receptive versus non‐receptive females (GLMM display_social‐treatment_: *χ*_df = 1_ = 0.15, *p* = 0.69; GLMM display_female‐status_: *χ*_df = 1_ = 0.02, *p* = 0.87; Figure 2b; Table 4). However, mixed‐sex reared males performed significantly more sneak attempts than male‐only reared males overall (GLMM sneak attempts_social‐treatment_: *χ*_df = 1_ = 6.60, *p* = 0.010; Figure 2c; Table 4). Post hoc analyses revealed that this difference was driven by a greater frequency of sneak attempts by mixed‐sex reared males toward non‐receptive females (estimate_male‐only vs. mixed_: −0.90 ± 0.35 SE, *z* = −2.60, *p* = 0.010; Figure 2b). In contrast, the frequency of sneak attempts toward receptive females did not differ significantly between treatments in post hoc analyses (estimate_male‐only vs. mixed_: −0.29 ± 0.35 SE, *z* = −0.83, *p* = 0.41; Figure 2c). Additionally, males from both social treatments showed no significant difference in the time spent chasing females, or in overall time spent chasing receptive and non‐receptive females (GLMM time chasing_social‐treatment_: *χ*_df = 1_ = 0.68, *p* = 0.41; GLMM time chasing_female‐status_: *χ*_df = 1_ = 1.58, *p* = 0.21; Figure 2d; Table 4).

**TABLE 4:** Statistical results from mixed models assessing male guppy behavior in no‐choice tests with non‐receptive and receptive females.

Trait	Parameter	IRR [95% CI]	SE	*z*	*p*
Frequency of display	Intercept	15.91 [10.93, 23.16]	3.05	14.45	**< 0.001**
	Female status (receptive)	1.04 [0.64, 1.69]	0.26	0.17	0.87
	Male social treatment (mixed‐sex)	1.10 [0.69, 1.75]	0.26	0.39	0.70
	Body size difference	1.08 [0.91, 1.29]	0.10	0.87	0.38
	Female gliding frequency	1.00 [0.96, 1.03]	0.02	−0.25	0.80
	Female status: male social treatment	1.08 [0.54, 2.14]	0.38	0.22	0.82
Frequency of sneak	Intercept	1.16 [0.67, 2.03]	0.33	0.53	0.60
	Female status (receptive)	1.64 [0.81, 3.33]	0.59	1.38	0.17
	Male social treatment (mixed‐sex)	2.45 [1.24, 4.87]	0.86	2.57	**0.010**
	Body size difference	0.90 [0.70, 1.15]	0.11	−0.83	0.40
	Female gliding frequency	1.05 [1.00, 1.10]	0.03	1.84	0.07
	Female status: male social treatment	0.54 [0.21, 1.42]	0.27	−1.25	0.21
Time spent chasing	Intercept	1363.9 [858.5, 2167.1]	322.2	30.56	**< 0.001**
	Female status (receptive)	1.44 [0.81, 2.55]	0.42	1.26	0.21
	Male social treatment (mixed‐sex)	1.28 [0.71, 2.30]	0.38	0.82	0.41
	Body size difference	0.85 [0.67, 1.09]	0.10	−1.29	0.20
	Female gliding frequency	1.01 [0.96, 1.05]	0.02	0.30	0.76
	Female status: male social treatment	0.71 [0.32, 1.56]	0.29	−0.85	0.39
	Zero Inflation: Intercept	0.04 [0.01, 0.11]	0.02	−6.29	**< 0.001**

Trait	Parameter	Coefficient [95% CI]	SE	*z*	*p*
Latency to first sexual behavior	Female status (receptive)	1.52 [0.81, 2.49]	0.47	1.38	0.17
	Male social treatment (mixed‐sex)	4.60 [2.19, 7.22]	1.58	4.43	**< 0.001**
	Body size difference	0.98 [0.78, 1.22]	0.12	−0.19	0.85
	Female gliding frequency	1.00 [0.96, 1.04]	0.02	−0.13	0.89
	Female status: male social treatment	0.41 [0.21, 1.04]	0.17	−2.09	**0.036**

*Note:* All models included female status (receptive vs. non‐receptive) and male social treatment (Mixed‐sex: *n* = 52; Male‐only: *n* = 53) as fixed effects, and their interaction, with random intercepts for experimental batch and rearing tank. Body size and female behavioral response were included as covariates. Generalized linear mixed models used a negative binomial distribution, with model estimates reported as incidence rate ratios (IRR): For latency to first sexual behavior, survival model estimates are reported as unstandardized coefficients. Significant *p*‐values (< 0.05) are shown in bold.

There was a significant interaction between female mating status and social rearing treatment in male latency to sexual behavior in no‐choice assays (survival model (latency to first behavior)_female‐status × social‐treatment_: *χ*_df = 1_ = 3.91, *p* = 0.048). Specifically, this interaction was driven by shorter time to perform sexual behaviors of mixed‐sex reared males with any female, although with the strongest differences observed when interacting with non‐receptive females (Non‐receptive_Mixed‐sex vs. Male‐only_: ∆ratio = 0.22 ± 0.07 SE, *z* = −4.34, *p* < 0.001, Receptive _Mixed‐sex vs. Male‐only_: ∆ratio = 0.54 ± 0.18 SE, *z* = −1.88, *p* = 0.06; Figure 2e; Table 4).

## 4 Discussion

We used dichotomous choice and no‐choice tests to investigate how social experience influences male sexual behavior and preference for female mating status in guppies. Males reared in mixed‐sex groups exhibited a higher frequency of coercive mating behaviors and initiated sexual activity more quickly with non‐receptive females compared to males reared in male‐only groups. Despite these observed adjustments in mating tactics, results from dichotomous choice and no‐choice assays were concordant in showing that our social treatments did not influence the overall strength of preference for receptive females.

The observed changes in mating tactics and shorter latency to initiate sexual behavior with non‐receptive females in mixed‐sex reared males highlight the important role of the social environment in shaping context‐dependent mating strategies. Our findings support previous work in guppies showing that males raised in mixed‐sex groups exhibit higher rates of coercive mating behaviors than those reared in male‐only groups, suggesting that the ability to strategically alternate between courtship and coercive tactics may be a learned process from interacting with females (Guevara‐Fiore 2012). Earlier studies have shown that male guppies typically increase coercive mating attempts toward non‐receptive females and sigmoid displays toward receptive ones (Guevara‐Fiore et al. 2010). In our study, this behavioral differentiation based on female mating status was only present in males reared in social environments with females, suggesting that feedback from interaction with females and experience with female receptivity cues is necessary for tactic adjustment. Together, these findings suggest that developmental exposure to females and variation in their receptivity flexibly modulate mating tactics and effort in response to female mating status. Such social experience may enhance reproductive efficiency in variable social environments. Comparable plasticity has been observed in a closely related species, *Poecilia wingei* (Endler's guppy), where exposure to varying levels of male–male competition modulated courting and coercive behavioral rates (Řežucha and Reichard 2014), further supporting the view that social cues shape male reproductive strategies.

Although our results show that social interaction with females shapes male mating behavior, our experimental design does not allow us to disentangle the specific mechanisms underlying this effect. One possibility is that prior experience with females improves males' ability to recognize which mating tactics provide higher success, as previously observed in guppies (Guevara‐Fiore and Endler 2018), and other species such as *Drosophila melanogaster* (Dukas 2005; Saleem et al. 2014; Balaban‐Feld and Valone 2017), and eastern mosquitofish (Bisazza et al. [1996] but see Iglesias‐Carrasco et al. [2019]). Alternatively, previous studies in guppies suggest that male mating strategies are shaped by variation in social conditions, including male–male competition or encounter rate with females (Corral‐López et al. 2020; Jirotkul 1999; Cattelan et al. 2016; Devigili et al. 2015; Jordan and Brooks 2012). It is therefore possible that differences in social dynamics between our treatment groups contributed to the behavioral divergence observed in our study. Regardless of the underlying mechanism, the tactic adjustments observed in males reared in mixed‐sex groups are consistent with theoretical predictions of fitness maximization, considering the lower energetic requirements of sneak insemination attempts compared to more costly sigmoid displays that yield higher success when female consent (Devigili et al. 2013). Thus, social experiences seem to modulate male mating behavior toward strategies that increase reproductive efficiency in variable social and ecological environments.

Despite previous findings that male guppies increase courtship rates and reduce coercive attempts toward receptive females over non‐receptive ones (Ojanguren and Magurran 2004; Romano and Stefanini 2021), we found no overall preference for receptive females across either of our social treatments. We consider it unlikely that our manipulation of female receptivity status failed, given it successfully elicited differential mating tactics in our no‐choice tests. The same protocol has been used in previous work, showing strong differences between receptive and non‐receptive treatments in female gliding approaches to the males, as confirmed in our experiment with positional data analyses (Guevara‐Fiore et al. 2010). A more plausible explanation is that laboratory conditions reduced ecological costs that normally shape preference expression in the wild (Kokko and Rankin 2006). In particular, the absence of ecological costs such as predation or food availability may have favored that in our experiment, males across treatments consistently invested in courtship displays far more frequently than in coercive mating attempts (see Figure 2). This imbalance suggests that in the absence of ecological constraints, males may default to the tactic with higher potential efficiency, even if more energetically demanding or with higher risks to be predated while performing it (Head et al. 2010; Magnhagen, 1991). In line with this, in other fish species, male courtship effort declines under resource limitation, while well‐fed males maintain high baseline courtship levels (Fernlund Isaksson et al. 2022; Olsson et al. 2009). Future work incorporating factors such as predation risk or food limitation should help determine the ecological relevance of social experience for male mating preferences.

Using both dichotomous and no‐choice approaches allowed for a broader picture of male preference variation influenced by social experiences. It is possible that mating preferences may be stronger in choice tests compared to no‐choice designs, as males can select the female that is more likely to result in insemination (Dougherty 2020). However, there is arguably an increased risk of being rejected by the only potential mate in a no‐choice test, and this could make males more careful in tuning their mate strategy to female receptivity cues (Dougherty and Shuker 2015). While our tests using these two complementary experimental paradigms were concordant in overall patterns of preference for receptive females across treatments, differences in tactics may still emerge depending on the context. For instance, our dichotomous assays incorporate female–female interaction effects, which may have influenced male decisions and that were absent in no‐choice designs. Thus, combining both approaches is valuable for integrating multiple dimensions of male mate choice.

The changes we observed in male guppies' behavioral repertoires and mating latency in response to female receptivity further support the important role of social environment and prior experience in modulating male sexual behavior. We found that social experience in mixed‐sex reared males did not alter the strength of preference for female receptivity status. However, the observed absence of preference for this trait in males tested prior to their social treatment assignment, combined with consistently high levels of energetically costly courtship behaviors across treatments, suggests that preference for female receptivity may be shaped by complex interactions with ecological factors in natural populations. For instance, predation risk, adult sex ratios, and their interaction effects have been shown to modulate the balance between display and coercive tactics in male guppies (Chuard et al. 2016, 2022; Godin 1995). Experimental integration of such ecological pressures with developmental social experience will be essential for further understanding the drivers of male mating strategies.

Overall, our findings support the idea that male guppies adjust their mating tactics when encountering receptive and non‐receptive females based on prior social experience. This observed behavioral flexibility highlights the importance of incorporating developmental and ecological context when assessing male mate choice, particularly in systems where multiple mating tactics coexist.

## Author Contributions

**Versara Goberdhan:** formal analysis (equal), investigation (lead), visualization (equal), writing – original draft (equal). **Wouter van der Bijl:** investigation (supporting), writing – review and editing (equal). **Iulia Darolti:** investigation (supporting), writing – review and editing (equal). **Judith E. Mank:** conceptualization (equal), resources (lead), supervision (supporting), writing – original draft (equal). **Alberto Corral‐Lopez:** conceptualization (equal), formal analysis (equal), supervision (lead), validation (lead), visualization (equal), writing – original draft (equal).

## Funding

This investigation was supported by NSERC and a Canada 150 Research Chair to J.E.M. A.C‐L. acknowledges personal support from the Birgitta Sintring Foundation (S2023‐0030 and S2025‐0026) and Stiftelsen P E Lindahls stipendiefond (Royal Swedish Academy of Sciences; LN2023‐0007).

## Conflicts of Interest

The authors declare no conflicts of interest.

## Figures and Tables

**FIGURE A2:**
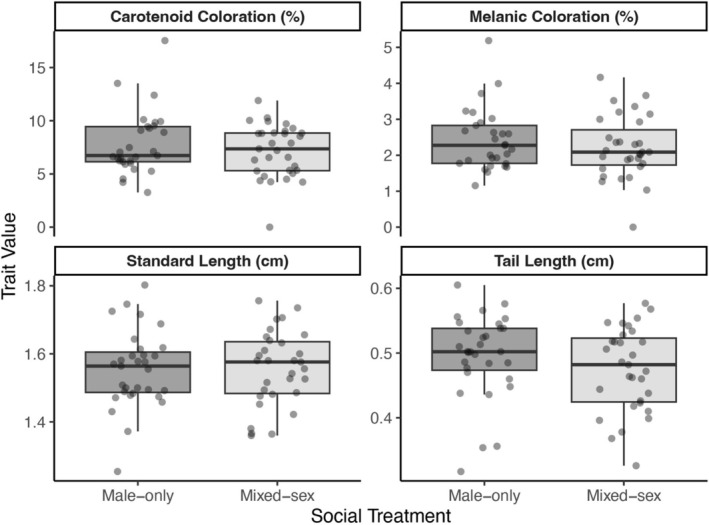
Body morphology and coloration in males used in experimental treatments. No significant differences were found between randomly assigned males in male‐only rearing (*n* = 31) and mixed‐sex rearing (*n* = 31) treatments for any of the coloration and morphological traits measured. For all boxplots, horizontal lines indicate medians, boxes indicate the interquartile range, and whiskers indicate all points.

## Data Availability

The datasets and analysis code supporting this study are available in a Figshare repository (DOI: https://doi.org/10.6084/m9.figshare.29264543).

## Appendix 1 Proximity‐Based Metrics as a Proxy of Preference

Prior to this study, we validated the use of male–female proximity as a proxy for sexual preference in guppies. For this, we analyzed a separate collection of video recordings from dichotomous choice trials in which one male interacted freely with two females in a circular arena (33 videos; 5‐min acclimation and 15‐min test). Both positional tracking and manual behavioral scoring were obtained for each trial. These trials were conducted in a separate laboratory using the same high‐predation Quare River stock as the current study (see Kotrschal et al. 2013). This work was part of an unrelated study on brain size and male sexual behavior (unpublished data).

Positional data were extracted using idTracker (Pérez‐Escudero et al. 2014) and used to quantify the time each male spent at various distances from each female. The number of courtship displays and coercive copulation attempts directed toward each female were scored by a single observer (A.C.‐L.) in idPlayer, which provided female identity information consistently throughout the recording (note that idTracker female id was uninformative of treatment).

Proximity‐based preference metrics were calculated as the proportion of time a male spent near each female across several distance thresholds. Likewise, a behavioral preference ratio was computed as the proportion of total sexual behaviors directed toward each female. We found the strongest positive correlation (*r* = 0.71, *p* < 0.001) between proximity‐based preference scores and behavioral preference ratios when using a threshold lower than 4 cm between male and female (Figure A1). These results support the use of proximity at this distance as a reliable proxy for male guppy preference in freely swimming dichotomous choice tests. The behavioral assays in the current study were designed to resemble the setup employed for this validation dataset, including experimental arena, lighting conditions, and trial duration.

## Appendix 2 Male Color and Morphology Analyses

We assessed coloration and morphology of males randomly assigned to mixed‐sex and male‐only rearing treatments. Average proportion of orange or black coloration did not differ between males assigned to these treatments (mean ± SE; orange coloration: male‐only reared males—7.83 ± 0.56, mixed‐sex reared males—7.14 ± 0.44, *F*_df = 1_ = 0.95, *p* = 0.33; black coloration: male‐only reared males—2.42 ± 0.15, mixed‐sex reared males—2.20 ± 0.15, *F*_df = 1_ = 0.99, *p* = 0.32; Figure A2). Similarly, there was no significant overall difference between males assigned to these experimental treatments in morphological traits (body size: male‐only reared males—1.55 ± 0.02, mixed‐sex reared males—1.56 ± 0.02, *F*_df = 1_ = 0.00, *p* = 0.95; tail size: male‐only reared males—0.49 ± 0.01, mixed‐sex reared males—0.47 ± 0.01, *F*_df = 1_ = 1.52, *p* = 0.22; Figure A2).
